# Encouraging understanding or increasing prejudices: A cross-sectional survey of institutional influence on health personnel attitudes about refugee claimants' access to health care

**DOI:** 10.1371/journal.pone.0170910

**Published:** 2017-02-14

**Authors:** Cécile Rousseau, Youssef Oulhote, Mónica Ruiz-Casares, Janet Cleveland, Christina Greenaway

**Affiliations:** 1 Department of Social and Cultural Psychiatry, McGill University, Montreal, Quebec, Canada; 2 Harvard T.H. Chan School of Public Health, Harvard University, Boston, Massachusetts, United States of America; 3 Research Centre of the University Institute with Regard to Cultural Communities, CIUSSS Centre-Ouest de l’Ile de Montreal, Montreal, Quebec, Canada; 4 Department of Epidemiology and Biostatistics, McGill University, Montreal, Quebec, Canada; Queensland University of Technology, AUSTRALIA

## Abstract

**Background:**

This paper investigates the personal, professional and institutional predictors of health institution personnel's attitudes regarding access to healthcare for refugee claimants in Canada.

**Methods:**

In Montreal, the staff of five hospitals and two primary care centres (n = 1772) completed an online questionnaire documenting demographics, occupation, exposure to refugee claimant patients, and attitudes regarding healthcare access for refugee claimants. We used structural equations modeling to investigate the associations between professional and institutional factors with latent functions of positive and negative attitudes toward refugee's access to healthcare.

**Results:**

Younger participants, social workers, participants from primary care centres, and from 1st migrant generation had the lowest scores of negative attitudes. Respondents who experienced contact with refugees had lower scores of negative attitudes (B = -14% standard deviation [SD]; 95% CI: -24, -4%). However, direct contact with refugees increased scores of negative attitudes in the institution with the most negative attitudes by 36% SD (95% CI: 1, 71%).

**Interpretation:**

Findings suggest that institutions influence individuals’ attitudes about refugee claimants’ access to health care and that, in an institutional context of negative attitudes, contact with refugees may further confirm negative perceptions about this vulnerable group.

## Introduction

Public representations of refugees in North America and Europe have progressively shifted from vulnerable individuals deserving protection to potential criminals and fraudsters seeking to abuse host country resources, in particular the health care system [1]. In parallel, there have been initiatives to welcome refugees such as the recent resettlement of 25,000 Syrian refugees in Canada. While the right to health for all, including vulnerable migrants, has been promoted by the United Nations, [2] governments have increasingly restricted health care coverage for precarious status migrants [3].

As the health impact of these policy changes on refugees is beginning to be documented, [4] it becomes evident that the obstacles to health care access related to these transformations are multilayered and should be analyzed within a systemic framework. Gee & Ford [5] showed how immigration policy influences health disparities both by modulating direct access through the introduction of different levels of coverage and through indirect obstacles stemming from racism manifesting as xenophobia. The racialization of immigration and the intersection of race, ethnicity or religion with socioeconomic and migratory status may constitute an important obstacle to health care access given that all of these factors have been shown to unconsciously bias clinicians, sometimes affecting their practices [6–9].

Research on attitudes of healthcare professionals towards immigrants and refugees is scant. Some studies suggest wide disparities between health professionals who favor a humanitarian or human rights perspective and those who feel they have the duty to protect their country’s scarce resources against what they understand as fraud and abuse [10–12]. Professional and personal background have been shown to be determinants of these attitudes [12,13]. There is however a dearth of studies examining the possible influence of institutional factors on professional attitudes, in spite of the growing evidence that institutional racism plays a role in health inequalities, [14] and that leadership and governance are central elements of a right to health approach [15].

In 2012, a major policy change restricted healthcare coverage for refugee claimants in Canada, introducing different levels of limited coverage [16]. We undertook a study to document its impact on systemic barriers to refugee claimants’ access to healthcare. Although full coverage of refugee healthcare was restored on April 1, 2016, actual access to care may vary depending on the attitudes of healthcare providers and institutions. The main aim of this paper is to answer the following research questions:

1. What are the personal, professional and institutional predictors of health institution personnel’s attitudes to access to healthcare for refugee claimants?
2. To what extent does direct contact with refugee claimants have a modifying effect on these attitudes?

## Methods

### Study population

The survey was conducted in Montreal, a large multiethnic city receiving about a quarter of the refugee claimants (aka asylum seekers) arriving in Canada. All administrators, clinicians, and other staff in five hospitals and two primary care centres, were invited to complete an online questionnaire. The five hospitals selected constitute all but one of the major general hospitals of Montreal, plus one (out of two) large pediatric hospital. Several of the hospitals are networks, so the five hospitals actually represent 12 locations and provide services to over 80% of the population of the Island of Montreal. The two primary care centres comprise 5 public primary care clinics out of a total of 29 on the Island of Montreal. They are located in areas with a high proportion of recent immigrants and refugee claimants.

### Survey design and administration

Refugee claimants were defined as individuals who apply for refugee status in Canada until either they are accepted as refugees or they are deported following the definitive rejection of their refugee claim. They are legally in Canada and entitled to Interim Federal Health (IFH) Program coverage. Government Assisted and Privately Sponsored Refugees eligible for provincial health insurance upon arrival were not considered in the study.

The survey instrument was designed by a multidisciplinary team specialized in refugee health and policy, translated (from English to French) and pretested. The wording of the items of the KAP survey was based on three main sources: First, a previous survey conducted by the first author on access to health care for pregnant women and children with precarious status in 2010–2011 validated the positive and negative attitude constructs [12]. Second, themes emerging from the open-ended comments in response to the previous survey on healthcare coverage for precarious status women and children were also taken into account [10]. This played a minor role in the formulation of survey items but confirmed the pertinence of the questions and their content validity. Third, analysis of the discourse of the Canadian government justifying the cuts to the IFHP and of the opponents to these cuts also provided qualitative data to validate the items [17]. The wording of the arguments for and against healthcare coverage for refugee claimants reflect the themes and wording adopted by advocates and opponents of cuts to refugee healthcare coverage in the two years immediately preceding the survey (2012–2014). The attitude questions elicit the most common positive and negative representations around these issues in the health sector. The survey contained 18 multiple-choice questions, recording respondents’ (a) demographics, occupation, institution and exposure to refugee claimant clients; (b) attitudes regarding healthcare access for refugee claimants; (c) knowledge about applicable federal and provincial healthcare coverage policies, (d) practices regarding access to services for refugee claimants. Questions about attitudes toward refugee claimants’ access to publicly funded health care were divided into two sections. The first concerned level of agreement on restricting access based on five arguments: cost, increased wait times, bogus refugee claims, taxpayer-only access to free healthcare and refugees take advantage by coming to receive free health care. The second section measured level of agreement with maintaining or expanding refugee claimants’ access to health care based on five arguments: compassion, legality of refugee claimant status, increased cost of delayed health-seeking, refugees as future citizens, and access to healthcare as a fundamental human right. All the answers were recorded on a 4-point Likert scale. Two questions documented respondent direct seeking health care and frequency of contact with culturally diverse population. Full text of survey questions is in supplemental material.

The questionnaire was administered using LimeSurvey, over a period of six weeks in May-June 2014. A link to the survey was posted on the institutions’ intranet and/or emailed to respondents and individual email reminders were sent twice during the survey period.

Montreal hospitals have two internal institutional email lists, one including all physicians practicing at the institution and another including all employees (professional and non-professional), except for kitchen and maintenance staff and personal care attendants.

Participation was informed, voluntary, and anonymous (to preserve anonymity institutions are numbered). The Research Ethics Boards of all participating institutions approved the study. The overall response rate was 6.2% (Hospital 1:3.2%; Hospital 2: 3.3%; Hospital 3: 10.2%; Hospital 4: 15.5%; Hospital 5: 7.0%; Primary care centers: 16.9%.

### Statistical analyses

We considered the following predictors in the models as described in Table 1: age, gender, language, institution, generation, previous contact with refugees, and contact with patients from different cultural backgrounds. We selected the covariates retained in the final models *a priori* using Directed Acyclic Graphs (DAGs) [18] to infer a minimal set of sufficient confounders. Contact with refugees was forced in all the models to isolate the independent effect of the covariates regardless of the contact with refugees. Final models included age, occupation, institution, generation, and contact with refugees.

**Table 1:** Individual and institutional characteristics of the study population.

Characteristic	n	%
**Gender**		
Male	406	22.9
Female	1361	76.8
Other	5	0.3
**Age**		
29 or younger	213	12
30–39	454	26.5
40–49	463	26.1
50–59	481	27.1
60 or over	161	9.1
Generation		
1^st^	409	23.3
2^nd^	364	20.7
3^rd^ (both parents born in Canada)	985	56.0
Missing	14	-
Occupation		
Medical Doctor	334	18.9
Nurse	324	18.4
Social worker	116	6.6
Other professional	157	8.9
Administrative employee	463	26.3
Manager	206	11.7
Other	163	9.2
Missing	9	-
Type of institution		
Hospital	1362	76.9
Primary care centre	410	23.1
Contact with refugee claimant		
No	913	51.5
Yes	859	48.5
Contact with culturally diverse population		
≥ 1 per week	1352	76.4
≤ 1 per week	417	23.6
Missing	3	-

**Fig 1:**
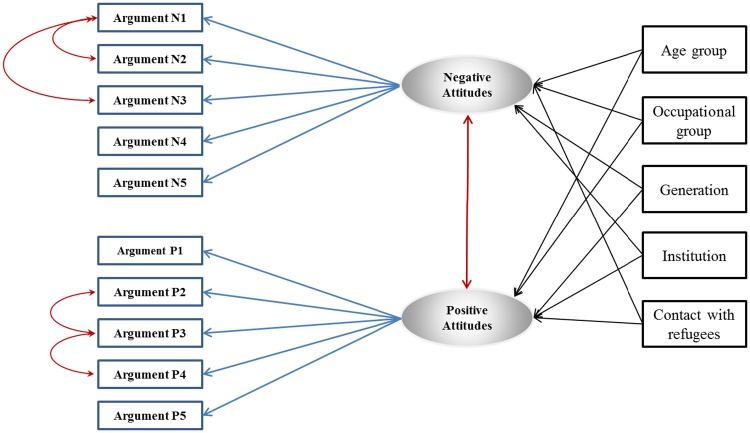
Conceptual path diagram of factors associated with latent constructs of negative and positive attitudes. Observed variables (measured) are represented by a rectangle, whereas latent (unmeasured) factors are represented by an ellipse. Single headed arrows are used to define a causal path, whereas double-headed arrows indicate covariances and correlations, without a causal link. The latent function of negative attitudes was indicated by reported levels of agreement on restricting access to health care for RC based on the 5 arguments (Argument N1: Cost; Argument N2: increased wait times; Argument N3: bogus refugee claims; Argument N4: taxpayer-only access to free healthcare; Argument N5: refugees take advantage by coming to receive free health care). The latent function of positive attitudes was indicated by reported levels of agreement on maintaining or expanding access to health care for RC based on the 5 arguments (Argument P1: compassion; Argument P2: legality of refugee claimant status; Argument P3: increased cost of delayed health-seeking; Argument P4: refugees as future citizens; Argument P5: access to healthcare as a fundamental human right).

We used structural equations modeling (SEM) [19] to investigate the association between professional and institutional predictors and attitudes towards access to health care. SEM is a powerful technique that combines a path analysis with a confirmatory factor analysis. It includes a measurement part in which the observed outcomes are linked to a limited number of latent functions, and a structural part describing the relationship between the latent variables and other observed variables [20]. This approach is often used in the case of theoretical constructs (in our case, measuring attitudes), and allows reduction of multiple comparisons testing and a more comprehensive assessment of negative and positive attitudes. We considered responses to individual questions regarding specific attitudes toward access to health care for refugee claimants as indicators of two underlying broader latent functions reflecting positive and negative attitudes (Fig 1). The positive attitudes function was indicated by the reported levels of agreement with maintaining or expanding refugee claimants’ access to publicly funded health care based on five arguments, whereas the negative attitudes function was indicated by reported levels of agreement on restricting refugee claimants’ access based on five arguments. We constructed these latent variables using a confirmatory factor analysis (CFA), allowing good discriminant validity. We also tested a model with a single construct including responses to the 10 questions as direct indicators to a latent function of positive attitudes (reversing the responses for the questions about negative attitudes). However, the overall model with one latent function showed a poor fit to the data and we decided to keep the two construct model. To correct for local dependence when the correlation between the indicators could not be fully explained by the underlying latent variable, we allowed measurement errors of several outcomes indicating the same latent function to correlate. Because the response modalities of the original outcome scores were ordinal (Likert scale), we used a weighted least squares mean- and variance-adjusted estimator, a robust estimator which provides better estimates for modeling categorical or ordinal data compared to the maximum likelihood estimator [21]. We tested goodness of model fit using several indices that are the most insensitive to sample size, model misspecification and parameter estimates [22]: the root mean square error of approximation (RMSEA), the comparative fit index (CFI), and the weighted root mean square residual (WRMR)

In additional analyses, we explored possible differences in the associations between individual and institutional characteristics and attitudes in regard to contact with refugees. We tested the difference in the associations among the groups with and without previous contact with refugees by comparing the value of *d*/SE_*d*_ to the standard normal distribution, where *d* is the difference between the two estimates, and $SE_{d}=\sqrt{SE_{1}^{2}+SE_{2}^{2}}$ is the standard error of the difference.[23]

All tests were two-sided, and analyses were conducted using the *lavaan* [24] package in R.[25]

## Results

### Descriptive analyses

The survey was completed by 1,772 participants. Table 1 describes the characteristics of the study population. Most participants were women (77%) and worked in hospitals (77%). Fifty two percent reported having previous contact with a refugee claimant, 56% were 3^rd^ generation (i.e. had both parents born in Canada), and 76% had regular contact with populations from different cultural backgrounds. Contact with refugees varied across occupational groups, with 61% of professionals, 36% of managers, 35% of administrative employees, and 33% of others indicating ever having had contact with a refugee claimant seeking healthcare (data not shown).

About 49% of respondents agreed on restricting refugee claimants’ access to health care based on the argument that refugee claimants take advantage of the Canadian health system, while 42%, 32%, and 37% of respondents agreed on restricting refugee claimants’ access to health care based on the arguments about cost, increased wait times and taxpayer-only access, respectively. Finally, only 23% of respondents agreed that access to health care should be restricted on the grounds that many refugee claims are bogus (Table 2).

**Table 2:** Respondent’s levels of agreement on restricting and maintaining/expanding refugee claimants’ access to publicly funded health care by argument.

Response	n	%	n	%	n	%	n	%	n	%
**Negative arguments**	**Cost**		**Wait times**		**Bogus claims**		**Taxpayers only**		**Taking advantage**	
Strongly disagree	476	27.0	612	34.7	741	42.1	628	35.7	423	24.1
Somewhat disagree	556	31.5	587	33.3	608	34.5	492	27.9	482	27.4
Somewhat agree	486	27.5	376	21.3	266	15.1	390	22.2	466	26.5
Strongly agree	247	14.0	189	10.7	146	8.3	251	14.3	388	22.1
Total	1765	100	1764	100	1761	100	1761	100	1759	100
**Positive arguments**	**Compassion**		**Legality**		**Delayed care**		**Future citizens**		**Human rights**	
Strongly disagree	60	3.4	104	5.9	96	5.5	46	2.6	57	3.3
Somewhat disagree	200	11.4	404	23	412	23.5	183	10.4	135	7.7
Somewhat agree	885	50.3	757	43	762	43.5	861	49.1	586	33.4
Strongly agree	613	34.9	494	28.1	480	27.5	664	37.9	976	55.6
Total	1758	100	1759	100	1750	100	1754	100	1754	100

Regarding positive attitudes, 89%, 87%, and 85% of respondents agreed on maintaining or expanding refugee claimants’ access to health care based on the arguments that healthcare is a human right, refugees may be future citizens, and compassion, respectively, and 71% agreed based on legal status and cost of delayed care (Table 2).

### Determinants of positive and negative attitudes

Table 3 describes the factor loadings and estimated correlation of measured variables to each of the constructed latent variables. All model fit indices showed an excellent fit to data (RMSEA = 0.04; CFI = 0.996; WRMR = 0.86). All observed variables for attitudes were adequate indicators of the latent functions with significant standardized correlations ranging between 0.68 and 0.93. The CFA also showed strong negative correlation between latent functions of positive and negative attitudes (*r* = -0.81).

**Table 3:** Factor loadings and estimated correlation of measured variables to latent functions.

Latent functions	Measured variable	Factor loading	SE	p-value	variance explained by the latent construct
Negative attitudes	Cost	1^a^	0	NA	93%
	Wait times	0.92	0.01	<0.001	85%
	Bogus claims	0.93	0.01	<0.001	86%
	Taxpayers only	0.87	0.01	<0.001	81%
	Taking advantage	0.94	0.01	<0.001	87%
Positive attitudes	Compassion	1^a^	0	NA	80%
	Legality	1.14	0.02	<0.001	91%
	Delayed care	0.85	0.02	<0.001	68%
	Future citizens	1.02	0.02	<0.001	81%
	Human rights	1.00	0.02	<0.001	80%

^a^ The latent function is constructed on the scale of the first component.

In multivariable models of negative attitudes (Table 4), social workers had scores of negative attitudes lower by -0.41 standard deviation [SD] (95% CI: -0.65, -0.18) compared to doctors. All other occupational groups had higher levels of negative attitudes compared to doctors with the highest ones being administrative employees (B = 0.65 SD; 95% CI: 0.50, 0.80). Respondents from primary care centres had scores for negative attitudes lower by -0.24 SD (95% CI: -0.40, -0.08) compared to respondents from hospital 1, whereas respondents from Hospital 5 had scores of negative attitudes higher by 0.24 SD (95% CI: 0.07, 0.42). Respondents aged 40–49 and respondents aged 50–59 years had scores of negative attitudes respectively higher by 0.37 SD (95% CI: 0.21, 0.53) and 0.29 SD (95% CI: 0.13, 0.46), compared to the youngest participants. Non-migrant and 2^nd^ generation immigrant respondents had scores of negative attitudes higher by 0.38 SD (95% CI: 0.26, 0.49) and 0.26 SD (95% CI: 0.12, 0.39) respectively compared to 1^st^ generation migrant respondents. Finally, respondents with previous contact with refugees had scores lower by -0.14 SD (95% CI: -0.24, -0.04) compared to respondents with no previous contact with refugee claimants. Results regarding the latent variable of positive attitudes exhibited the opposite pattern (Table 5).

**Table 4:** Associations between the potential predictors and scores of the latent variable of negative attitudes.

	Standardized estimate	lower 95% CI	upper 95% CI	p-value
**Occupational group**				
Doctors	Reference	-	-	-
Nurses	0.31	0.15	0.48	0
Social workers	-0.41	-0.65	-0.18	0
Other professionals	0.19	0	0.39	0.05
Managers	0.36	0.17	0.54	0
Administrative employees	0.65	0.5	0.8	0
Others	0.62	0.44	0.81	0
**Institution**				
Hospital 1	Reference	-	-	-
Hospital 2	0.09	-0.08	0.26	0.31
Hospital 3	0.05	-0.1	0.19	0.55
Hospital 4	-0.01	-0.2	0.18	0.91
Hospital 5	0.24	0.07	0.42	0.01
Primary care centres	-0.24	-0.4	-0.08	0
**Age**				
29 or younger	Reference	-	-	-
30–39	0.12	-0.04	0.28	0.15
40–49	0.37	0.21	0.53	0
50–59	0.29	0.13	0.46	0
60 or older	0.05	-0.16	0.26	0.62
**Generation**				
1st generation	Reference	-	-	-
2nd generation	0.26	0.12	0.39	0
3^rd^ (Both parents born in Canada)	0.38	0.26	0.49	0
**Contact with refugees**				
No	Reference	-	-	-
Yes	-0.14	-0.24	-0.04	0.01

**Table 5:** Associations between the potential predictors and scores of the latent variable of positive attitudes.

	Standardized estimate	lower 95% CI	upper 95% CI	p-value
**Occupational group**				
Doctors	Reference	-	-	-
Nurses	-0.19	-0.37	-0.02	0.03
Social workers	0.31	0.07	0.55	0.01
Other professionals	-0.26	-0.47	-0.05	0.02
Managers	-0.28	-0.49	-0.08	0.01
Administrative employees	-0.49	-0.65	-0.33	0
Others	-0.61	-0.82	-0.4	0
**Institution**				
Hospital 1	Reference	-	-	-
Hospital 2	-0.2	-0.39	-0.02	0.03
Hospital 3	-0.05	-0.21	0.11	0.53
Hospital 4	-0.07	-0.28	0.14	0.5
Hospital 5	-0.32	-0.51	-0.13	0
Primary care centres	0.14	-0.03	0.31	0.11
**Age**				
29 or younger	Reference	-	-	-
30–39	-0.24	-0.41	-0.07	0.01
40–49	-0.37	-0.55	-0.2	0
50–59	-0.38	-0.56	-0.21	0
60 or older	-0.18	-0.4	0.04	0.11
**Generation**				
1st generation	Reference	-	-	-
2nd generation	-0.12	-0.27	0.03	0.12
3^rd^ (Both parents born in Canada)	-0.29	-0.42	-0.17	0
**Contact with refugees**				
No	Reference	-	-	-
Yes	0.09	-0.01	0.2	0.09

**Fig 2:**
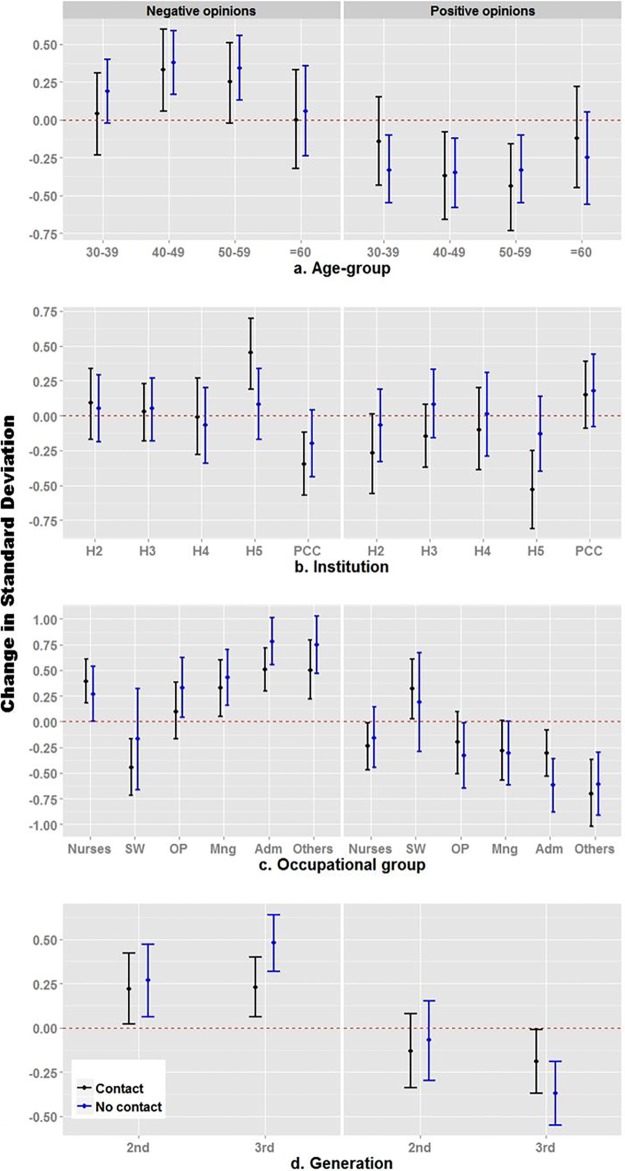
Factors associated with latent constructs of negative and positive attitudes, stratified by contact with claimants.

In further analyses exploring the modification effect by contact with refugee claimants, we observed significant effect modification by contact with refugees (p<0.05) in the associations between institution and generation, and negative attitudes regarding refugee claimants’ access to care (Fig 2). Regarding institutions, there were no apparent differences in negative attitudes between respondents with and without previous contact with refugee claimants in hospitals 2, 3, and 4. Most importantly, Hospital 5 and primary care centres exhibited opposite patterns, with contact with refugees significantly increasing negative attitudes in Hospital 5 (*d* = 0.36; SE_*d*_ = 0.18), while decreasing negative attitudes in primary care centres (*d* = -0.15; SE_*d*_ = 0.17), but not significantly. Likewise, previous contact with refugee claimants attenuated negative attitudes in respondents from the 3^rd^ generation (*d* = -0.25; SE_*d*_ = 0.12). Previous contact with refugee claimants attenuated negative attitudes in most occupational groups except nurses, though not significantly (Fig 2).

We observed similar patterns for positive attitudes, with previous contact with refugee claimants increasing positive attitudes among administrative employees (*d* = 0.31; SE_*d*_ = 0.17) and social workers. Likewise, contact with refugee claimants lowered positive attitudes in respondents from Hospital 5 (*d* = -0.40; SE_*d*_ = 0.20).

## Discussion

The main findings from this investigation are that in the Montreal health sector, personal, professional and institutional factors influence positive and negative attitudes of healthcare professionals toward refugee claimants’ entitlement to healthcare. In terms of individual characteristics, age and migration history have a significant influence. Younger and older healthcare personnel appear to be more sensitive to the refugee predicament. In institutions where overall attitudes were either particularly negative or positive, direct contact with refugee claimants amplified the dominant institutional tendency. The modification effect size was larger than the effect size of contact with refugee claimants itself. In Hospital 5, the institution where negative attitudes were most prevalent, contact was significantly associated with more negative attitudes than the absence of contact. Conversely, in primary care centres, where more positive attitudes towards healthcare access for refugee claimants predominated, respondents reporting previous contact with refugee claimants had more positive attitudes than those who did not, although not significantly. Contact did not modify attitudes in institutions where attitudes were less polarized.

Our findings converge with previous studies on the topic. The fact that first and second generation immigrants have more positive attitudes toward refugee claimants’ access to health care is consistent with the results of a similar survey about undocumented patients in Montreal. This may possibly be related to less prejudice toward refugees and more identification with migrants’ experience [26].

As in this previous study, profession was also associated with attitudes and had the strongest observed effect sizes, with social workers having the most positive attitudes and administrative personnel among the most negative (Tables 4 and 5). This could be, in part, associated with the level of contact and engagement with refugee claimants. In particular, contact with refugees was associated with a decrease in negative attitudes among most of the occupational groups, especially social workers and administrative employees, although the effect modification did not reach the level of significance.

Social cognition literature emphasizes that individual attitudes are influenced by the implicit and explicit prejudices and stereotypes that prevail in the person’s social environment, including the workplace, [27,28], as well as by institutional policies [29]. The presence of outgroup members (here, refugee claimants) is often initially experienced as threatening and may lead to increased hostility, but positive interpersonal contacts between ingroup and outgroup members have been consistently found to help in overcoming prejudice and promoting friendly relations [30]. Negatively valenced contacts, however, may increase prejudice [31]. Our findings are overall in line with this trend.

The differences among institutions, and in particular the fact that primary care centre personnel showed more positive attitudes than hospital personnel, even after adjustment for contact with refugees, is consistent with findings of a study on undocumented patients [12]. Our qualitative data and our knowledge of the different institutions can shed some light on the contact-institution relationship. All participating institutions service the highly multiethnic population of the Island of Montreal. However, they do not share the same historical relations with migrant and refugee communities. The participating primary care centres have traditionally welcomed vulnerable newly arrived families who settle first in the low-cost housing neighborhoods they service. They often, in different ways, have advocated institutionally for this population. Among the hospitals, some are situated in the central part of Montreal, while notably Hospital 5, are in neighborhoods that were not traditionally migrant neighborhoods, and for which this may be a relatively new reality. Further research is needed to document to what extent a tradition of care and advocacy for refugee patients and/or the actual leadership of an institution are associated with staff attitudes toward entitlement to health care for these groups.

A recent study found that individual attitudes toward outgroup members are more positive when living in social contexts in which people have, on average, more cordial intergroup contact, with group contact having an even larger impact on reduction of prejudice than individual contact [32]. This may be due to more tolerant attitudes being perceived as normative. Our results converge with these observations suggesting that the predominant values and stereotypes in an institution may frame individuals’ experience of the encounter with refugee claimants and that, in a context of overall negative representations; contact may further confirm preexisting prejudices.

These results have implications for institutional and health systems decision makers. By highlighting the importance of institutional variables as determinants of attitudes, they lend support to the hypothesis of institutional leadership and orientation as an obstacle or a facilitator to health care access for refugee claimants. They indicate that to address the institutional stance toward vulnerable or excluded populations and raise awareness around the potential bias of majority in decision-making, [33] the active support of the highest levels of the institution may be needed [29]. Dealing with vulnerable immigrant exclusion from healthcare requires a systemic conceptualization of the issue to address the upsurge in negative representations of refugees [5,34].

Some limitations should be noted. Variation in the means used for recruitment in different institutions (i.e., staff and physicians’ email lists, intranet) may explain variance in response rates by institution and professional groups. Response rates were generally low and may have been influenced by strength of opinions on the issue under study, thus limiting the generalizability of results. The measure of prior contact is limited as it does not evaluate frequency or valence [35]. We have no information as to whether respondents considered their contact with either refugee claimants or with ‘culturally diverse populations’ as negative or positive. The literature has increasingly shown that attitudes are predicted not only by the amount of previous intergroup contact, but also by its valence [31,32,35–37]. As we do not have data on valence of contact, this greatly limits our ability to interpret the role of contact.

## Conclusions

This study provides evidence that beyond health professional and administrative staff’s personal and professional positions, institutions may have a significant role as facilitators or obstacles to vulnerable migrants’ access to health care. While more research is warranted to understand the processes associated with this institutional influence, this indicates that institution leadership should be a focus of action to promote more equity in health care access and that training and rights education need to raise awareness about personal and institutional biases [38,39].

## Supporting information

S1 Table Survey responses. The file contains the anonymized survey results (full data set).
(XLS)
